# Weibel Palade Bodies: Unique Secretory Organelles of Endothelial Cells that Control Blood Vessel Homeostasis

**DOI:** 10.3389/fcell.2021.813995

**Published:** 2021-12-16

**Authors:** Johannes Naß, Julian Terglane, Volker Gerke

**Affiliations:** Centre for Molecular Biology of Inflammation, Institute of Medical Biochemistry, University of Muenster, Muenster, Germany

**Keywords:** calcium, exocytosis, lysosome-related organelle, secretory organelles, hemostasis

## Abstract

Vascular endothelial cells produce and release compounds regulating vascular tone, blood vessel growth and differentiation, plasma composition, coagulation and fibrinolysis, and also engage in interactions with blood cells thereby controlling hemostasis and acute inflammatory reactions. These interactions have to be tightly regulated to guarantee smooth blood flow in normal physiology, but also allow specific and often local responses to blood vessel injury and infectious or inflammatory insults. To cope with these challenges, endothelial cells have the remarkable capability of rapidly changing their surface properties from non-adhesive (supporting unrestricted blood flow) to adhesive (capturing circulating blood cells). This is brought about by the evoked secretion of major adhesion receptors for platelets (von-Willebrand factor, VWF) and leukocytes (P-selectin) which are stored in a ready-to-be-used form in specialized secretory granules, the Weibel-Palade bodies (WPB). WPB are unique, lysosome related organelles that form at the trans-Golgi network and further mature by receiving material from the endolysosomal system. Failure to produce correctly matured VWF and release it through regulated WPB exocytosis results in pathologies, most importantly von-Willebrand disease, the most common inherited blood clotting disorder. The biogenesis of WPB, their intracellular motility and their fusion with the plasma membrane are regulated by a complex interplay of proteins and lipids, involving Rab proteins and their effectors, cytoskeletal components as well as membrane tethering and fusion machineries. This review will discuss aspects of WPB biogenesis, trafficking and exocytosis focussing on recent findings describing factors contributing to WPB maturation, WPB-actin interactions and WPB-plasma membrane tethering and fusion.

## Introduction

Endothelial cells comprise the inner lining of blood vessels and thus the first cellular barrier separating blood and tissue. They form single-layered epithelia that differ in morphology, molecular characteristics, physiology and function depending on the type of vascular bed. As such they seal blood vessels and control traffic of nutrients, hormones, growth and differentiation factors, particles and cells (immune cells, metastasizing tumor cells and even pathogens) to and from the vasculature. Moreover, through selective secretion and uptake as well as production and decoding of signaling molecules they regulate blood vessel homeostasis including clotting and coagulation, fibrinolysis and thrombosis as well as vascular tone and local inflammatory reactions.

One striking characteristic of endothelial cells relates to the adhesive properties of their apical cell surface that faces the blood vessel lumen. In the normal physiological state this surface does not interact firmly with leukocytes, erythrocytes and platelets thereby permitting an unrestricted blood flow and blood cell circulation. However, upon insult and endothelial cell activation surface properties change rapidly allowing leukocytes and platelets to adhere to the vessel wall. These cell interactions are vital to ensure proper responses to blood vessel injury (platelet plug formation and initiation of coagulation) and inflammatory or infectious insult (recruitment of leukocytes to sites of tissue damage or infection). Endothelial cells can actively control these surface properties by the regulated presentation of specific adhesion molecules. To do so, vascular endothelial cells are equipped with unique secretory organelles that store among other things leukocyte and platelet adhesion receptors to be released on demand. In honor of their initial discovery by Ewald Weibel and George Palade in electron microscopic analyses of rat and human pulmonary arteries these organelles were termed Weibel-Palade bodies (WPB) (Weibel and Palade, 1964). Only later these peculiar membrane compartments were shown to contain the major platelet adhesion molecule von-Willebrand factor (VWF) and the leukocyte receptor P-selectin (Wagner et al., 1982; Bonfanti et al., 1989). The physiological and also pathophysiological importance of WPB and their principal cargo VWF is emphasized by the fact that failure to produce and release proper VWF results in von-Willebrand disease, the major inherited bleeding disorder (for reviews see Schneppenheim and Budde, 2011; Leebeek and Eikenboom, 2016). On the other hand, vascular occlusion is a consequence of highly elevated vascular VWF levels as for instance observed in thrombotic thrombocytopenic purpura. (for review see Sadler, 2008). Thus, WPB are pivotal components of the precisely tuned machinery that orchestrates blood vessel homeostasis. This mini review will highlight the unique features of WPB particularly emphasizing recent developments in the understanding of their maturation and secretion.

### WPB Maturation

WPB are born at the trans-Golgi network (TGN) where they bud off in the form of discernible structures. Their dimensions and unique morphology are dictated by the main cargo VWF, a large glycoprotein synthesized and first processed in the ER (for references and recent crystal structure of the VWF D’D3 domains see Dong et al., 2019). VWF is then transported to the Golgi where it is assembled into defined quanta. A copacking of these quanta occurs in the TGN prior to or concomitant with the actual budding of immature WPB which can maintain connections to the Golgi for 2–4 h (Zenner et al., 2007; Ferraro et al., 2014; Mourik et al., 2015). These connections and the close proximity to the Golgi likely permit the further addition of VWF and possibly other cargo to the immature WPB (Mourik et al., 2015). The early WPB released from the TGN further mature to finally yield the highly elongated cigar-shaped organelles primarily found in the periphery of endothelial cells (for reviews see van Mourik et al., 2002; Michaux and Cutler, 2004; McCormack et al., 2017; Karampini et al., 2020). This maturation is driven on one hand by the continued multimerization and tight packing of VWF into a quasi-crystalline arrangement enwrapped by a membrane, which requires luminal acidification and reflects itself in the condensation of WPB from an electron lucent immature organelle to an electron dense mature structure. On the other hand, post-Golgi maturation is accompanied by acquisition of additional cytosolic and also endosomal/lysosomal components. They include the RabGTPase Rab27a and the tetraspanin CD63 identifying WPB as lysosome-related organelles (LRO) that share molecular features with pigment-storing melanosomes (for reviews see Raposo et al., 2007; Bowman et al., 2019). It is worth noting here that the net size of WPB is primarily determined at the level of the Golgi and that further maturation mainly leads to condensation and tubular elongation. Several aspects of WPB size control and maturation have been addressed recently revealing novel and exciting connections.

**FIGURE 1:**
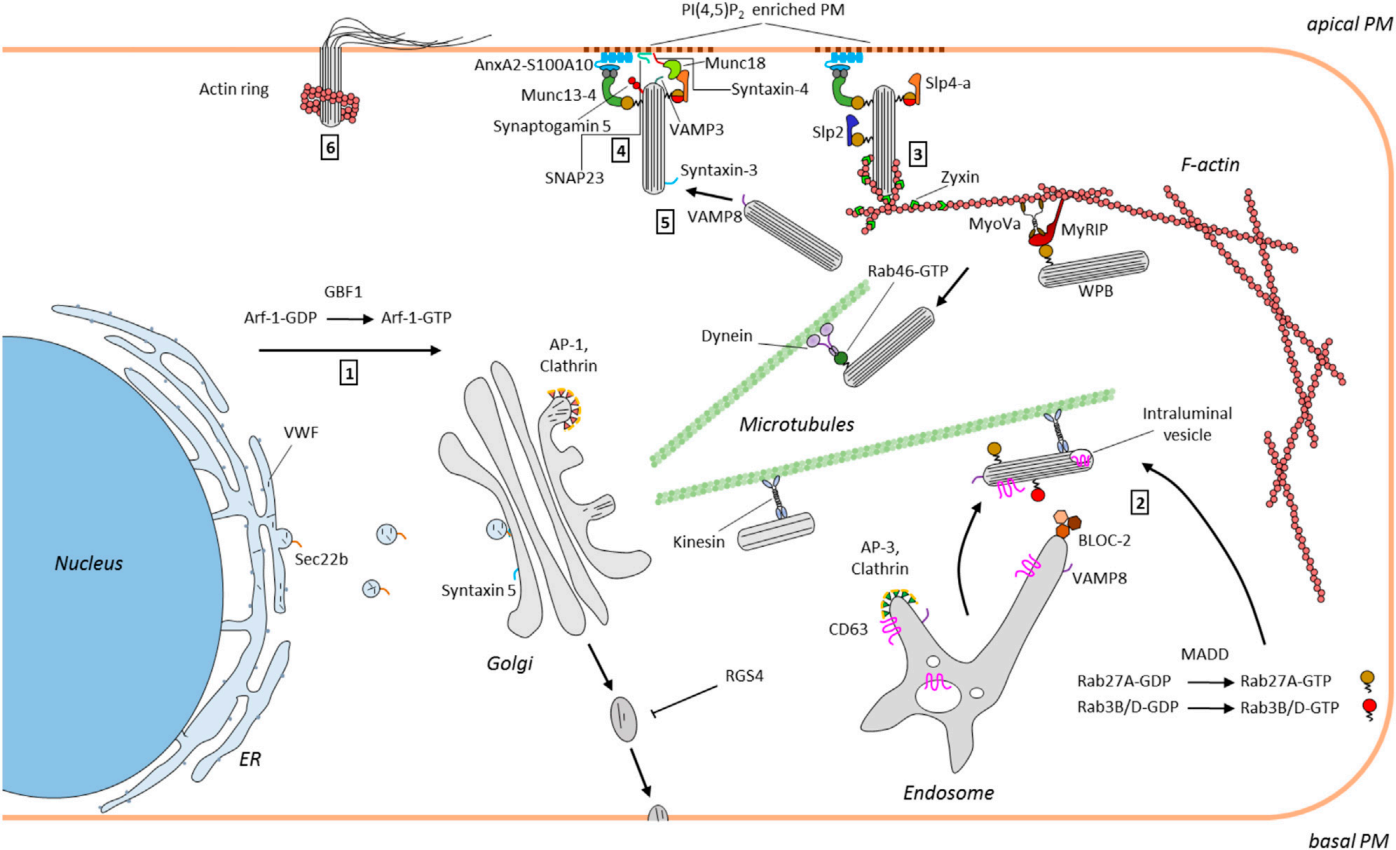
Scheme depicting the WPB itinerary in endothelial cells. WPB formation is driven by VWF that is produced at the ER and trafficked to the Golgi (1). WPB that bud from the TGN in an AP-1 and clathrin dependent process are then transported to the cell periphery along microtubules. This is accompanied by the BLOC-2 and annexin A8 dependent transfer of endosomal components such as CD63 and VAMP8 to WPB (2). Maturing WPB acquire certain RabGTPases, e.g. Rab27A and Rab3B/D, the former required for linking WPB at the cortical actin cytoskeleton (via MyRIP/MyoVc) and supporting exocytosis (*via* Slp4-a) (3). Secretagogue induced tethering at and fusion with the PM requires docking factors, such as the annexin A2/S100A10/Munc13-4 complex and a SNARE-based fusion machinery and can also involve compound and cumulative events (3, 4 and 5). Finally, post fusion actin rings have been observed that support the full release of highly multimeric VWF (6). Mainly factors identified in the recent years have been included.

An interesting link between WPB size control and cell metabolism was discovered recently following the identification of the Arf guanine nucleotide exchange factor (GEF) GBF1 (a GEF for Arf1 and 4) as a factor promoting ER/Golgi trafficking of VWF. GBF1 can be activated by phosphorylation by AMP-activated protein kinase (AMPK), a key enzyme coupling metabolic changes to cellular signaling, and it was shown that low glucose levels and subsequent AMPK activation lead to GBF1 phosphorylation and a resulting upregulation of anterograde VWF trafficking. This in turn produces smaller WPB and reduces VWF secretion (Lopes-da-Silva et al., 2019) (Figure 1). Arf GTPase activating proteins (GAPs) that inactivate their cognate Arf proteins also appear to regulate WPB size as depletion in endothelial cells of the ArfGAP SMAP1 leads to a size reduction in the WPB that form (Watanabe et al., 2021). The SNARE Sec22b was recently identified as another factor controlling WPB morphology presumably also by affecting the ER/Golgi transport route of VWF. Depletion of Sec22b causes a loss of large, elongated WPB along with a dilation of ER cisternae that accumulate non-processed VWF (Karampini et al., 2020) (Figure 1). Thus, several factors regulating VWF maturation and packing into WPB and thereby affecting WPB size and morphology have been discovered and approaches to exploit these also in pathophysiological settings appear promising. Along these lines, Ferraro and coworkers developed a microscopic screening approach measuring WPB size that led to the identification of first candidate compounds that reduce WPB length. As a consequence, this also reduces the pro-thrombotic activity of secreted VWF as VWF secretion from shorter WPB significantly dampens its platelet adhesion capability (Ferraro et al., 2016, 2020).

Once early WPB have emerged from the Golgi they acquire additional proteins (and presumably also lipids) in the process of maturation that is accompanied by a microtubule-dependent movement to the cell periphery (for review see McCormack et al., 2017). Many of those additional WPB components have been identified, among other things through proteomic screens (van Breevoort et al., 2012; Holthenrich et al., 2019); however, their delivery to and association with the maturing organelle has only recently been characterized in a few cases. In line with WPB representing LRO, some proteins found on mature WPB are of late endosome/lysosome (LEL) origin (e.g. the tetraspanin and P selectin cofactor CD63) and most likely routed to the organelle by direct transport possibly involving tubular carriers. Whereas earlier studies had identified the Ca^2+^/phospholipid-binding protein annexin A8 as a LEL-localized component of the machinery facilitating LEL-to-WPB delivery of CD63 (Poeter et al., 2014), Sharda and coworkers (Sharda et al., 2020) recently reported the participation of biogenesis of lysosome related organelle-2 (BLOC-2), a protein that can be mutated in the recessive bleeding disorder Hermansky-Pudlak syndrome. Among other things Hermansky-Pudlak syndrome is associated with platelet aggregation and pigmentation defects, the latter due to compromised maturation of melanosomes, LROs that show several parallels to WPB (for reviews see Raposo et al., 2007; Simons and Raposo, 2009). Depletion of BLOC-2 results in both, compromised LEL-to-WPB transport of CD63 and general WPB maturation defects with the WPB appearing round instead of elongated and clustered in the perinuclear region (Figure 1). As the immature organelles formed under these conditions failed to process VWF into the highly multimeric forms these were absent in the material secreted from BLOC-2 depleted endothelial cells following thrombin stimulation (and intracellular Ca^2+^ mobilization). Moreover, the exocyst complex was identified as a target of BLOC-2 in endothelial cells and exocyst depletion or inhibition phenocopied the WPB maturation defects seen in BLOC-2 deficient cells. In this study exocyst was also found to serve a second function in impeding WPB exocytosis at the PM (Sharda et al., 2020). The involvement of BLOC-2 in proper WPB maturation was also shown in the respective mutant mice that are characterized by impaired VWF tubulation (Ma et al., 2016). Another gene that can be mutated in Hermansky-Pudlak syndrome is *AP3B1* encoding the adaptor complex three *β*1 subunit. Blood outgrowth endothelial cells from Hermansky-Pudlak syndrome patients carrying the *AP3B1* mutation also lack CD63 in their WPB indicative of improper organelle maturation. Moreover, these cells are compromised in their evoked WPB exocytosis, most likely because they fail to recruit the v-SNARE VAMP8 to maturing WPB (Karampini et al., 2019) (Figure 1). While the above-mentioned studies identified maturation factors/pathways involved in the delivery of transmembrane proteins (CD63, VAMP8) to maturing WPB, another hallmark of mature WPB are a specific subset of cytosolically associated RabGTPases, in particular Rab27a and the Rab3 isoforms b and d. Addressing this aspect of the maturation, Kat and coworkers (Kat et al., 2021) could recently identify MAP kinase-activating death domain (MADD) as a crucial component involved. MADD serves as a GEF for these Rabs and silencing of MADD through knockdown approaches markedly reduced the recruitment of Rab27a, Rab3b and Rab3d to maturing WPB (Figure 1). Finally, it should be noted that WPB maturation is not only accompanied by tubulation and tight packing of VWF and the acquisition of additional protein contents, it also generates other morphological characteristics typical for LRO. Specifically, vesicles inside the lumen of the organelle, a hallmark of many LRO, were observed recently in mature WPB of endothelial cells. Following WPB exocytosis these intraluminal vesicles which are positive for CD63 could also be released and possibly function in intercellular communication, again extending the similarity to other LROs (Streetley et al., 2019).

Thus, WPB maturation is a highly complex process involving *de novo* protein acquisition, LEL-to-WPB protein transport and morphological alterations that eventually generate the unique rod-shaped organelle containing the tubulated highly multimeric VWF.

### WPB-Plasma Membrane Tethering and Secretion

The VWF stored in WPB can be released in different ways. Basal secretion, typically of less multimeric VWF, provides the circulation with low levels of these VWF species, and constitutive secretion, preferentially occurring at the basolateral membrane surface of endothelial cells, deposits VWF in the subendothelial matrix. Specific components regulating these secretory events have not been systematically investigated with the exception of a recent screen that identified the regulator of G protein signaling 4 (RGS4) as a negative regulator of the constitutive pathway (Patella and Cutler, 2020). The majority of fully matured WPB, however, is retained inside the cell to await secretagogue stimulation, for example following blood vessel injury or local inflammatory insults, to present highly multimeric VWF and P-selectin on the endothelial cell surface by regulated exocytosis. Retention is achieved by anchorage in the cortical actin cytoskeleton, which is mediated with help of a complex consisting of Rab27a, the Rab27a effector MyRIP and the actin binding myosin Va (Nightingale et al., 2009; Rojo Pulido et al., 2011; Conte et al., 2016) (Figure 1). Endothelial stimulation, which can be elicited by a plethora of agonists (Lowenstein et al., 2005; Schillemans et al., 2019b) and typically results in elevated intracellular Ca^2+^ or cAMP levels functioning as second messengers, mobilizes the cortically anchored WPB and initiates the tethering/docking at and fusion with the plasma membrane (PM). The detailed molecular mechanisms responsible for releasing WPB from the cortical anchorage and enabling their PM contact are largely unknown, but they are likely to involve WPB associated RabGTPases. A central role for Rab27a in this event has been shown by Bierings and coworkers (Bierings et al., 2012) who reported that the evoked release of mature WPB is regulated by the interaction of Rab27a with either MyRIP (supporting cortical anchorage) or synaptotagmin-like protein 4-a (Slp4-a) (promoting WPB exocytosis) (Figure 1). Rab46 was recently identified as another Rab regulating selective WPB trafficking in the cell cortex and thereby specific cargo release following histamine evoked and Ca^2+^ mediated exocytosis of WPB. Rab46, which harbors a Ca^2+^-binding EF hand, localizes to only a subset of the peripheral WPB that are negative for the leukocyte receptor P-selectin but contain angiopoietin-2. It senses the Ca^2+^ elevation elicited by histamine stimulation and then triggers a retrograde, dynein-dependent transport of the associated peripheral WPB to the cell center. As the Rab46 negative, P-selectin containing WPB exocytose under these conditions, only a fraction of the WPB cargo, e.g. the proinflammatory P-selectin, is released (Miteva et al., 2019) (Figure 1). How and when such WPB diversification, i.e. a sorting of P-selectin to only some organelles, occurs and how Rab46 is recruited to only a subset of WPB is not known but these pose interesting and very central cell biological questions.

Following cortical release and in preparation of PM fusion, WPB are most likely tethered or docked at the membrane. Here, another Rab27a effector, the mammalian uncoordinated 13–4 (Munc13-4), has been shown to promote WPB exocytosis most likely by providing a link or tether between the organelle surface and a PM-bound complex consisting of annexin A2 (AnxA2) and S100A10 (Zografou et al., 2012; Chehab et al., 2017) (Figure 1). In this configuration the AnxA2/S100A10 complex most likely functions as a module binding Ca^2+^-dependently to PM phospholipids [e.g. phosphatidylinositol 4,5-bisphosphate, PI(4,5)P_2_] via its AnxA2 subunit and to WPB-bound Munc13-4 via its S100A10 subunit (Chehab et al., 2017). A special enrichment of certain PM phospholipids is indeed observed at WPB-PM fusion sites and inhibitor and depletion experiments suggest that PI(4,5)P_2_ and the PI(4,5)P_2_ producing PI4P 5-kinase are required for efficient histamine-evoked WPB exocytosis (Nguyen et al., 2020). In the course of regulated exocytosis tethered WPB are finally recognized by the membrane fusion machinery consisting of SNAREs and associated proteins. Several of the factors involved at this stage have been described over the years, including a trans-SNARE complex consisting of WPB-localized VAMP3 and PM-localized syntaxin-4 and SNAP23 as well as syntaxin-binding Munc18 proteins (Matsushita et al., 2003; Pulido et al., 2011; van Breevoort et al., 2014) (Figure 1). However, the picture is probably more complex as recent studies employing blood outgrowth endothelial cells which were isolated from a patient suffering from variant microvillus inclusion disease and shown to lack another SNARE, syntaxin-3, showed markedly impaired agonist-evoked VWF secretion. Syntaxin-3 interacts with VAMP8, another WPB-associated v-SNARE, but interestingly, was shown to localize mainly to WPB (Schillemans et al., 2018, 2019). This suggests that syntaxin-3, most likely pairing with VAMP8 on another WPB, supports homotypic fusions of WPB that could occur during compound or cumulative exocytosis (Zupančič et al., 2002; Valentijn et al., 2010; Kiskin et al., 2014; Stevenson et al., 2017) (Figure 1). Thus, several SNARE complexes are likely to support heterotypic and homotypic WPB fusion events that characterize the final steps in regulated exocytosis. Common to these events is their regulation by signaling mediators, in the case of Ca^2+^-dependent exocytosis the elevated Ca^2+^ concentrations. Several Ca^2+^ binding proteins have been implicated in coupling these Ca^2+^ signals to regulated WPB exocytosis, including the above-mentioned Slp4-a, AnxA2 and Munc13-4 as well as another Munc13 family member, Munc13-2 (Zhou et al., 2016; Holthenrich et al., 2019); however, the actual WPB-associated Ca^2+^ sensor that could directly activate the SNARE machinery most likely is a member of the synaptotagmin family. Synaptotagmin-5 has recently emerged as an interesting candidate as it localizes to WPB and is required for histamine evoked WPB exocytosis and VWF secretion. Importantly, a mutant synaptotagmin-5 lacking the Ca^2+^ coordinating asparagine residue in the C2A domain negatively interferes with histamine evoked WPB exocytosis directly showing the importance of synaptotagmin-5 Ca^2+^ binding (Lenzi et al., 2019). Thus, a complex interplay of Ca^2+^-regulated proteins, also including the recently identified Slp2-a (Francis et al., 2021), likely transmits the rise in intracellular Ca^2+^ to WPB-PM docking and fusion in the course of regulated exocytosis.

### The Link to Actin

While cargo release in many exocytotic events occurs automatically with completion of the granule-PM fusion, WPB and some other secretory organelles carrying large cargo, e.g. surfactant-loaded lamellar bodies of alveolar epithelial cells (Miklavc et al., 2015), most likely require mechanical forces for efficient cargo expulsion. This can be provided by rearrangements of the cortical actin cytoskeleton that first has to be weakened to allow granule penetration to the PM and then site-specifically repolymerizes to support cargo release. In the case of WPB, it was observed that rings of polymerized actin form at the distal end of WPB several seconds after the actual PM fusion event (Figure 1). Furthermore, it was shown that these structures, in an active myosin motor-dependent process, are required for the efficient release of highly multimeric VWF cargo from the fused WPB (Nightingale et al., 2011). In later studies it was observed that the formation of such actin rings at WPB-PM fusion sites probably is not obligatory for VWF release, at least in case of histamine stimulation and Ca^2+^-dependent WPB exocytosis (Conte et al., 2015), and that the extent of actin ring formation at these fusion sites appears to depend on the type of stimulus (Nightingale et al., 2018; Mietkowska et al., 2019). Interestingly, a different actomyosin network that is also positive for the focal adhesion protein zyxin has been observed around peripheral WPB of endothelial cells stimulated with cAMP raising agonists. Here, actin framework formation occurs prior to the actual fusion event facilitating WPB exocytosis (Han et al., 2017; Li et al., 2018). Clearly, more work is required to establish a potential link between this zyxin/actomyosin network and the post-fusion actin rings, e.g. by identifying the factor(s) promoting actin polymerisation into the ring/coat-like structures at fused WPB. Moreover, the precise function of the actin structures also needs further attention. They could support exocytotic membrane fusion and VWF expulsion but potentially could also prevent fused WPB from fully collapsing into the PM, for example to permit rapid and spatially restricted compensatory endocytosis that has been shown to occur on the membrane of fused WPB (Stevenson et al., 2017). Another unresolved issue concerns the regulation of the spatially restricted changes in cortical actin architecture, in particular the questions whether certain membrane lipids enriched at WPB fusion sites such as PI(4,5)P_2_ are involved and which molecular players organize the actin reorganization precisely at the sites where WPB fuse or have fused.

### Concluding Remarks

WPB are unique secretory organelles that allow vascular endothelial cells to respond rapidly to environmental changes by the secretion of factors that control hemostasis and inflammation. Marked progress in understanding their biogenesis, intracellular transport and secretion has been made in the last decade revealing fascinating cell biological phenomena that drive the formation of the organelle and its many modes of exocytosis. However, our picture of the organelle is far from complete and important questions, e.g. concerning unique maturation steps, cargo selection and Rab recruitment and the involvement of different actin structures in VWF release, remain to be answered. Future research in this exciting topic of cell biology has to tell and will likely also benefit pharmacological interventions of the pathway that could help controlling vascular VWF (and P-selectin) levels in pathophysiological scenarios (Karampini et al., 2020; El-Mansi and Nightingale, 2021).
